# How Can a Deep Learning Algorithm Improve Fracture Detection on X-rays in the Emergency Room?

**DOI:** 10.3390/jimaging7070105

**Published:** 2021-06-25

**Authors:** Guillaume Reichert, Ali Bellamine, Matthieu Fontaine, Beatrice Naipeanu, Adrien Altar, Elodie Mejean, Nicolas Javaud, Nathalie Siauve

**Affiliations:** 1 Radiology Department, Louis Mourier Hospital, Assistance Publique-Hôpitaux de Paris (APHP), University of Paris, 92700 Colombes, France; contact@alibellamine.me (A.B.); fontainematthieu@yahoo.fr (M.F.); abeatricen@yahoo.com (B.N.); 2 Emergency Department, Louis Mourier Hospital, AP-HP, 92700 Colombes, France; dr.adrien.altar@gmail.com (A.A.); nicolas.javaud@aphp.fr (N.J.); 3 Emergency Department, Foch Hospital, 92150 Suresnes, France; Elodie.mejean@gmail.com; 4 INSERM, U970, Paris Cardiovascular Research Center—PARCC, 75015 Paris, France

**Keywords:** deep learning, fracture detection, medical images, convolutional neural networks, conventional X-rays, medical application of deep learning

## Abstract

The growing need for emergency imaging has greatly increased the number of conventional X-rays, particularly for traumatic injury. Deep learning (DL) algorithms could improve fracture screening by radiologists and emergency room (ER) physicians. We used an algorithm developed for the detection of appendicular skeleton fractures and evaluated its performance for detecting traumatic fractures on conventional X-rays in the ER, without the need for training on local data. This algorithm was tested on all patients (*N* = 125) consulting at the Louis Mourier ER in May 2019 for limb trauma. Patients were selected by two emergency physicians from the clinical database used in the ER. Their X-rays were exported and analyzed by a radiologist. The prediction made by the algorithm and the annotation made by the radiologist were compared. For the 125 patients included, 25 patients with a fracture were identified by the clinicians, 24 of whom were identified by the algorithm (sensitivity of 96%). The algorithm incorrectly predicted a fracture in 14 of the 100 patients without fractures (specificity of 86%). The negative predictive value was 98.85%. This study shows that DL algorithms are potentially valuable diagnostic tools for detecting fractures in the ER and could be used in the training of junior radiologists.

## 1. Introduction

One of the principal reasons for attending the emergency room (ER) is peripheral traumatism. The first radiological examination in any case of suspected fracture remains the conventional X-ray. Fractures can be difficult for junior physicians to diagnose in situations of high patient flux [1,2]. The misdiagnosis of fractures directly affects patient management, and serious complications, such as malunion or arthritis, may occur if fractures are diagnosed late or remain undiagnosed. Fracture misdiagnosis is also one of the commonest causes of litigation in the domain of medicine. 

Deep learning (DL) is a subfield of machine learning relating to algorithms inspired by the structure and function of the brain, known as artificial neural networks. DL is a subfield in the domain of machine learning in artificial intelligence, in which networks are capable of learning from data in an unsupervised manner. Since 2012, DL has established itself as the cutting-edge method of enhancing performance in medical image analysis, with the use of convolutional neural networks decreasing the classification error rate from about 25% in 2011 to 3.6% in 2015 [3,4]. This success has led to numerous applications in medicine, for identifying and classifying images for diabetic retinopathy [5], and for detecting skin cancer [6] or lesions on mammograms [7].

Fracture detection on conventional X-rays in the ER remains challenging, particularly given the continually high patient flow through the ER, 24 h per day, and the reliance on junior radiologists for front-line diagnosis.

A new generation of software based on DL technology is emerging as a way of facilitating fracture diagnosis and preventing misinterpretation [8]. This technology could be used in the design of triage tools [9] for use in the ER. Several algorithms and methods [10] have been developed and are being evaluated. High levels of performance, with sensitivities and specificities greater than 90%, have been reported [11,12], paving the way for future applications in diagnostic assistance. A recent study [13] on retrospectively selected wrist X-rays showed that the performance of clinicians, and possibly also junior radiologists, was improved by reading standard X-rays in conjunction with a fracture detection algorithm. 

Artificial intelligence algorithms are opening up many new perspectives for radiologists. They save time, provide diagnostic assistance, and, for inexperienced doctors, can provide a learning tool for the reading of standard X-rays [13].

The objectives of this study were to evaluate the performance of a DL algorithm for the diagnosis of extremity fractures in a consecutive series of adult patients consulting at the emergency department of Louis Mourier and to define its potential uses in clinical practice.

## 2. Materials and Methods

### 2.1. Algorithm

The algorithm is an ensemble algorithm composed of multiple object detection models. 

**Figure 1:**
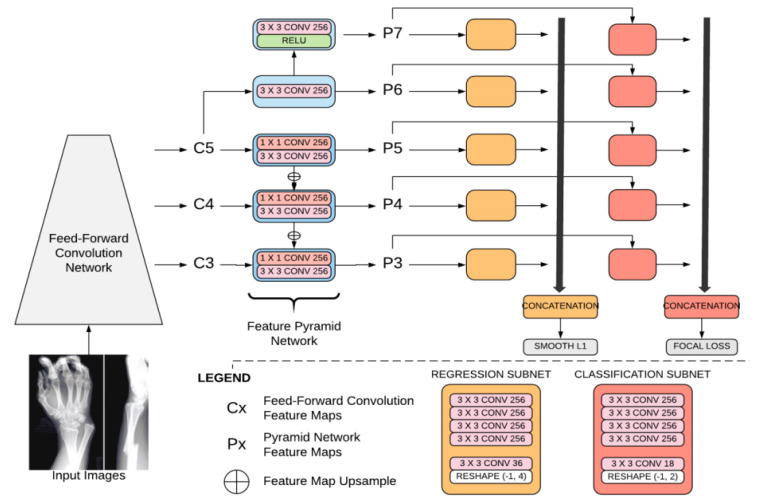
Diagram of the algorithm (adapted from the documentation of the algorithm company). Each object detection model outputs a list of bounding boxes (this list can be empty if no fracture is detected by the object detection model) with their corresponding confidence scores. A majority vote between the predicted bounding boxes of the models is used to select the final list of bounding boxes. The difference between the object detection models in the ensemble is their weight initializations.

Each object detection model is based on artificial convolutional neural network (ACNN) technology and, more specifically, is derived from the architecture of RetinaNet [14], an open-source DL algorithm. It has three components. The first is a custom backbone (based on the VGG neural network [15] but with fewer filters and batch normalization performed before each convolution) that acts as a feature extractor. This backbone consists of convolutional layers, max-pooling layers, and trainable batch normalization layers. The second component is a feature pyramid network (FPN) designed to extract the features at different resolutions, given the large variability of fracture size. The final component is two subnetworks; a classification subnet for predicting the presence or absence of a fracture, and a regression subnet for localizing the site of the fracture more precisely. The classification subnet predicts the probability of an object being present, for any class (two classes in our case), at each spatial position, for each anchor. The classification subnet is applied to each pyramid level, but the parameters of this subnet are shared across all pyramid levels. The classification subnet is a fully convolutional network. The regression subnet predicts the offset from each anchor box to a nearby ground-truth object (if such an object exists). We also applied this regression subnet to each pyramid level (with shared weights). This subnet is also a fully convolutional network. It is similar to the classification subnet except that, rather than predicting two (i.e., the number of classes) values, it has four values per anchor (Figure 1). 

RetinaNet optimizes two losses during training:

1. Focal loss: Focal loss is a cross-entropy with a modulating factor with a gamma parameter. This parameter affects the loss such that easy-to-classify samples are down-weighted in the classification loss.
2. A smooth L1 loss (such as regression loss), used to bound regression boxes. A smooth L1 loss is less sensitive to outliers than the L2 loss. The batch size is 4. The network was regularized during training, based on weight decay (L2). As the outline of a fracture is subjective, this loss has been smoothed for the purposes of fracture detection.

The total loss focuses more on the classification loss than the regression loss, as the goal is to help the radiologist to identify fractures on X-rays.

The algorithm was trained from scratch with Adam optimizer [16] with a learning rate of 1.0 × 10^−4^ over 50 epochs, with a halving of the learning rate if no progress was made in terms of validation loss for three epochs. The model was evaluated on the AUC value. During training, data augmentation was used to transform the images randomly. The transformations used were horizontal and vertical flipping, rotation, zooming, and shifting. 

Neural networks recognize fractures through supervised learning. Images are annotated for a pattern to be identified, in this case, the presence and location of a fracture on an X-ray. Dedicated software (Medeye, Azmed) is used to delineate the boundaries of the fracture. The dataset generated in this manner is used to train the algorithm. The trained algorithm can then detect the presence of a fracture, which it identifies by drawing a box around it.

This algorithm is commercially available (Azmed, Paris, France) and has obtained the CE mark level IIA for medical devices. It was trained on 21,138 fractures on X-rays at five medical imaging centers and more than 10,000 X-rays without fractures. The performance of this algorithm has never been tested and reported in a peer-reviewed publication.

### 2.2. Dataset

From the 2958 patients consulting the Louis Mourier ER from 1 to 31 May 2019, two emergency physicians (AA and EM) retrospectively selected all the patients included in the computerized clinical database (UrQual, McKesson, Irving, TX, USA) with respect to the following inclusion criteria: patient at least 16 years old, presenting at the emergency room of Louis Mourier Hospital (AP-HP) from 1 to 31 May 2019, for non-life-threatening traumatism, for whom X-rays were performed and the physician issued a diagnosis on patient discharge. The exclusion criteria were: visit to the ER for any non-traumatic cause, and visits for spinal traumatism. In total, the clinicians identified 125 patients. 

The X-rays were read by the radiologist (GR), with the Picture Archiving and Communication System widely used at AP-HP (PACS, Carestream, Health France, 93160, Noisy-le-Grand), blind to both clinical findings and final diagnosis. The gold standard was the final diagnosis delivered by the radiologist on the basis of clinical information. 

All the X-rays were rendered anonymous and exported from PACS (GR) in DICOM format. Each X-ray was then annotated by the radiologist, who drew a box around the fracture. The algorithm was then evaluated by comparing the radiologist’s annotation with the prediction delivered by the algorithm.

### 2.3. Statistical Analysis

The match between the algorithm’s prediction and the radiologist’s annotations was evaluated by calculating the Jaccard coefficient. This method has the advantage of preventing outliers from being considered true positives. We applied a similarity threshold of 0.02 for the Jaccard index. Predictions satisfying this criterion were considered true positives, with other predictions being considered false positives.

The primary outcome was the sensitivity per patient. If the algorithm identified the fracture on a single incidence, the classification was considered correct. The secondary outcomes were negative predictive value, specificity, and area under the curve (AUC). We also assessed the performance for each image.

The two main evaluations metrics that are being used to assess the performance of the algorithm are described below:

- Sensitivity measures the proportion of positives that are correctly identified. In the following formula, *TP* stands for true positive and *FN* stands for false negative. Patients with a fracture that is correctly identified are considered true positives, whereas patients with a fracture not identified by the algorithm are considered false negatives.
  (1) $$Sensitivity=\frac{TP}{FN+TP}$$
- Negative predictive value measures the proportion of individuals with negative test results who are correctly diagnosed. In the following formula, *TN* stands for true negative and *FN* stands for false negative. Patients without a fracture that are correctly classified are true negatives, whereas patients with a fracture who are identified by the algorithm as having no fracture are considered false negatives.
  (2) $$Predictive\;negative\;value=\frac{TN}{FN+TN}$$
  Sensitivity, specificity, negative predictive value (NPV), and area under the curve (AUC) were calculated with Python (Python Software Foundation) and scikit-learn (https://scikit-learn.org, June 2007, a free Python library).

## 3. Results

In total, the emergency physicians included 125 patients in this study. The traumatism considered concerned the hip in sixteen patients, the hand in twenty-eight, the shoulder in twenty-eight, the foot in twenty one, the knee in seven, the wrist in twenty one, and the elbow in four. 

Fractures were identified in 25 patients. Diagnoses did not differ between emergency physicians and the radiologist, all of whom identified the same patients as having fractures. There were seven foot fractures, seven hand fractures, five wrist fractures, two ankle fractures, one femur fracture, one clavicle fracture, and two shoulder fractures.

**Figure 2:**
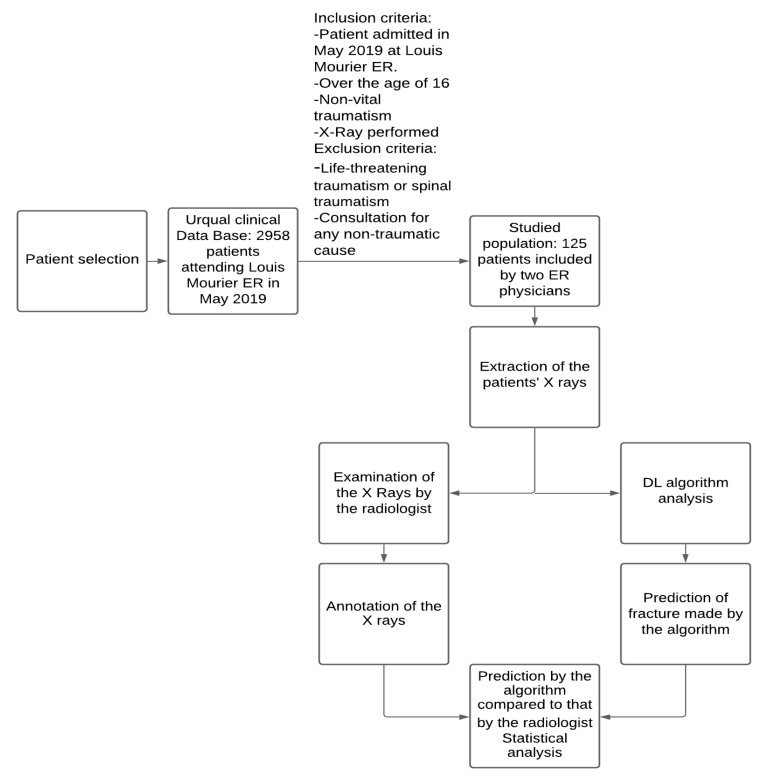
Constitution of the dataset.

**Figure 3:**
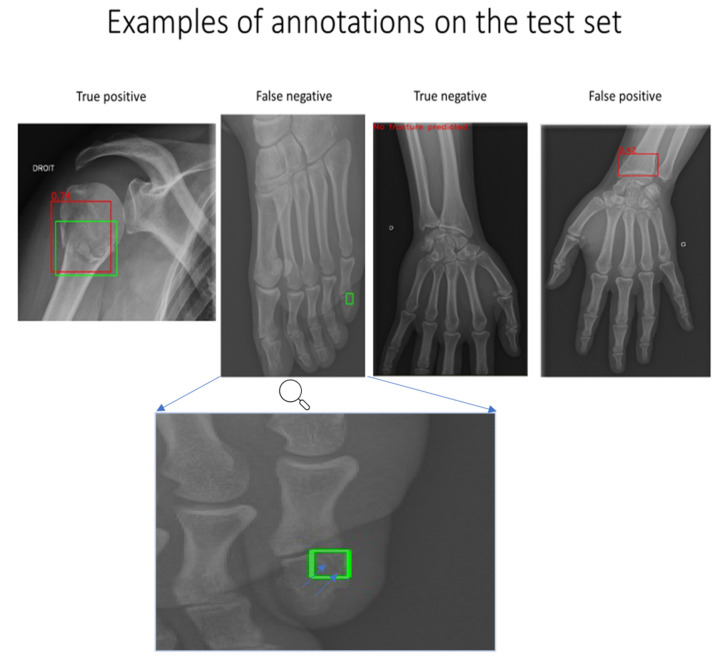
Example of predictions made by the algorithm (red square) versus annotations by the radiologist (green square). Blue arrows show the fracture.

**Figure 4:**
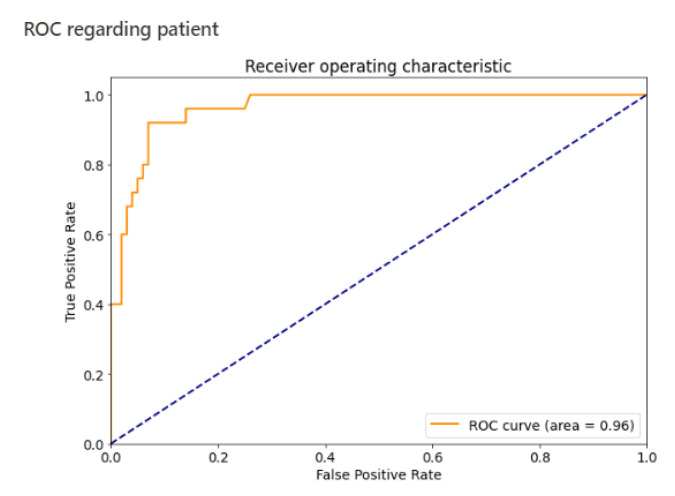
ROC curve, by patient.

The algorithm detected 24 of the 25 patients (Figure 2 and confusion matrix, Table 1) with fractures (a sample is shown in Figure 3). The fracture missed by the algorithm was a transverse fracture of the second phalanx of the left little toe. The algorithm also identified 14 patients as having fractures when they did not actually have a fracture. The sensitivity per patient was 96% (CI 95% 0.88–1) and the specificity per patient was 86% (CI 95% 0.79–0.93). The negative predictive value per patient was 98.85% (CI 95% 0.97–1). The area under the curve per patient was estimated at 0.96 (Figure 4).

**Figure 5:**
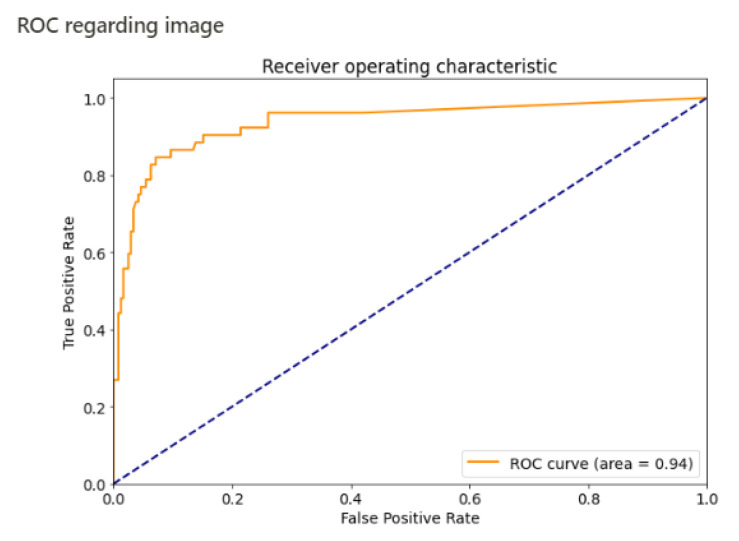
ROC curve, by image.

The sensitivity and specificity per image were 84% and 92%, respectively. The area under the curve per image was estimated at 0.94 (Figure 5). All the performances of the algorithm are summarized in Table 2.

## 4. Discussion

The objective of this study was to evaluate the potential added value of a DL algorithm for diagnosing fractures in the context of peripheral joint traumatism in an adult population attending the hospital ER. This study also provides the first evaluation of the feasibility and performance of the DL algorithm in this clinical situation.

For this first assessment, the algorithm diagnosed fractures with a sensitivity of 96%, a specificity of 0.86%, and a negative predictive value of 98.85%. Its performance in terms of sensitivity, our primary outcome, was similar to the values ranging from 83% [12] to 99% [17] reported in published studies. However, most of these studies focused on a single joint, or even on a specific type of fracture, whereas we evaluated the performance for a diverse set of peripheral skeletal fractures. However, the various studies performed to date are subject to certain limitations. 

Kim and MacKinnon [18] reported a sensitivity of 0.9 and a specificity of 0.88 for detecting wrist fractures, and Gan [19] also reported a sensitivity of 0.9 for detecting wrist fractures. Kitamura [20] reported a sensitivity of 0.86 for detecting pelvic fractures. Cheng [21] recently reported a sensitivity of 92% for detecting pelvic fractures. Jones [22] reported a sensitivity of 95.2% for detecting fractures throughout the peripheral skeleton, a performance close to that of the algorithm we tested.

We chose to evaluate the algorithm in the clinical context of patients attending the emergency department for traumatism, and to test its performance on various peripheral joints. A new generation of software based on DL technology is emerging to facilitate fracture diagnosis and prevent misinterpretation [8]. Fracture detection performance has increased over the last five years and is now very good. This progress can be partly explained by the continual development of faster, more efficient CNNs, making it possible to train the algorithm more rapidly and efficiently, using more data in less time. New approaches for identifying patterns on images as fractures are continually being developed, including object recognition, object detection (as in the tested algorithm), and segmentation. Most of the algorithms tested detect only one type of fracture, usually wrist fractures. Moreover, most of these algorithms have never been used in a clinical context, are not routinely used, and make use only of data imported from PACS without clinical information.

Most fractures of the peripheral skeleton were detected in our cohort of patients, and we found that this algorithm had a good negative predictive value (98%). This algorithm can therefore be used to identify patients without fractures with a high degree of confidence. This is one of the expectations of junior radiologists and the emergency physicians.

The fracture missed by the algorithm was a transverse fracture of the second phalanx of the left little toe. It remains unclear why the algorithm fails to detect certain fractures. The processing of the algorithm is opaque and cannot be understood directly, hence the notion of a “black box” commonly applied to such technology [23]. Several hypotheses can be put forward concerning the reasons for which the fracture of the second phalanx of the left little toe was missed. The first concerns the training set: an isolated fracture of the second phalanx is rarer than other foot fractures involving the distal phalanges or metatarsal bones. The algorithm was, therefore, probably less well-trained to deal with this type of fracture. The fracture was also difficult to see without modifying the contrast, probably due to underexposure of the image.

The false positives reflect the choice of the software developers to favor sensitivity and negative predictive values over the risk of a few false positives. A balance must be struck between detecting all fractures and the risk of overfitting: too many false positives for a small gain in detection. 

Our results are encouraging and suggest that this algorithm could provide clinicians with diagnostic assistance for fracture detection, and could be used as a learning tool for junior radiologists.

Moreover, this algorithm was easy to implement because it did not have to be trained on a training set from Louis Mourier Hospital for analysis of the data from this hospital. In most previous studies, local training was considered necessary to achieve a high level of performance [24,25]. The use of a large training set containing more than 20,000 X-rays of fractures, and X-rays from different centers probably overcomes the obstacles generally encountered in this field, such as differences in pixel distribution between X-ray machines. Our findings thus demonstrate the feasibility of domain adaptation, a subfield of DL. One of the classical limitations of DL is the amount of labeled data required to train the algorithm to perform a specific task in a specific data set. In our case, CNNs made it possible to apply a trained algorithm to a new data set on which it was not trained, such as the data from a new radiological center. Progress in the field of CNNs, with domain adaptation, may lead to many benefits for fracture detection, such as the absence of a need to adapt the algorithm for a new center, or much smaller data requirements for the training with less data to train the algorithm for a new center.

This study has several weaknesses: it was a retrospective study, and only a relatively small number of patients were included. The results are promising but require confirmation in prospective clinical studies. Other studies could also focus on the similarity or lack of similarity between the recognition of fractures by an expert human and the algorithm.

## 5. Conclusions

This first study on a population attending the ER for peripheral traumatism shows that a DL algorithm can be used, with a high level of accuracy, including a high negative predictive value in particular. A DL algorithm with no training could be used at a new center without the need for data from this site, for the diagnosis of fractures in a population of patients consulting for traumatisms of any peripheral joint.

Artificial intelligence algorithms are opening up many new perspectives for radiologists. They save time, provide diagnostic assistance, and, for inexperienced doctors, can provide a learning tool for the reading of standard X-rays. For clinicians, such algorithms can help to prevent errors, particularly during the night, and should improve the fracture detection performance of clinicians [13].

## Figures and Tables

**Table 1:** Confusion matrix describing the distribution of patients.

Results/Fracture Status	Fracture	No Fracture
Detection	24	14
No detection	1	86
Total	25	100

**Table 2:** Summary table describing the performance of the algorithm.

	Algorithm Performance for Detecting Patients with a Fracture	Algorithm Performance for Detecting Fractures Per Image
Sensitivity	96% (95% CI 0.88–1)	84%
Specificity	86% (95% CI 0.79–0.93)	92%
AUC	0.96	0.94
Negative predictive value	98.85% (95% CI 0.97–1)	

## Data Availability

The data is in the electronic patient record, not accessible to the public.

## References

[1] Moonen P.J., Mercelina L., Boer W., Fret T. (2017). Diagnostic error in the Emergency Department: Follow up of patients with minor trauma in the outpatient clinic. Scand. J. Traum. Resusc. Emerg. Med..

[2] Wei C.J., Tsai W.C., Tiu C.M., Wu H.T., Chiou H.J., Chang C.Y. (2006). Systematic analysis of missed extremity fractures in emergency radiology. Acta Radiol..

[3] Russakovsky O., Deng J., Su H., Krause J., Satheesh S., Ma S., Huang Z., Karpathy A., Khosla A., Bernstein M. (2015). ImageNet Large Scale Visual Recognition Challenge. Int. J. Comput. Vis..

[4] Lakhani P., Sundaram B. (2017). Deep Learning at Chest Radiography: Automated Classification of Pulmonary Tuberculosis by Using Convolutional Neural Networks. Radiology.

[5] Oh K., Kang H.M., Leem D., Lee H., Seo K.Y., Yoon S. (2021). Early detection of diabetic retinopathy based on deep learning and ultra-wide-field fundus images. Sci. Rep..

[6] Alzubaidi L., Al-Amidie M., Al-Asadi A., Humaidi A.J., Al-Shamma O., Fadhel M.A., Zhang J., Santamaría J., Duan Y. (2021). Novel transfer learning approach for medical imaging with limited labeled data. Cancers.

[7] Kleppe A., Skrede O.-J., De Raedt S., Liestøl K., Kerr D.J., Danielsen H.E. (2021). Designing deep learning studies in cancer diagnostics. Nat. Rev. Cancer.

[8] Kalmet P.H.S., Sanduleanu S., Primakov S., Wu G., Jochems A., Refaee T., Ibrahim A., Hulst L.V., Lambin P., Poeze M. (2020). Deep learning in fracture detection: A narrative review. Acta Orthop..

[9] Berg H.E. (2017). Will intelligent machine learning revolutionize orthopedic imaging?. Acta Orthop..

[10] Kandel I., Castelli M., Popovič A. (2020). Musculoskeletal Images Classification for Detection of Fractures Using Transfer Learning. J. Imaging.

[11] Yoon A.P., Lee Y.-L., Kane R.L., Kuo C.-F., Lin C., Chung K.C. (2021). Development and Validation of a Deep Learning Model Using Convolutional Neural Networks to Identify Scaphoid Fractures in Radiographs. JAMA Netw. Open.

[12] Olczak J., Fahlberg N., Maki A., Razavian A.S., Jilert A., Stark A., Sköldenberg O., Gordon M. (2017). Artificial intelligence for analyzing orthopedic trauma radiographs: Deep learning algorithms—Are they on par with humans for diagnosing fractures?. Acta Orthop..

[13] Lindsey R., Daluiski A., Chopra S., Lachapelle A., Mozer M., Sicular S., Hanel D., Gardner M., Gupta A., Hotchkiss R. (2018). Deep neural network improves fracture detection by clinicians. Proc. Natl. Acad. Sci. USA.

[14] Lin T.Y., Goyal P., Girshick R., He K., Dollar P. (2020). Focal Loss for Dense Object Detection. IEEE Trans. Pattern Anal. Mach. Intell..

[15] Simonyan K., Zisserman A. Very deep convolutional networks for large-scale image recognition. Proceedings of the 3rd International Conference on Learning Representations, ICLR.

[16] Kingma D.P., Ba J.L. Adam: A method for stochastic optimization. Proceedings of the 3rd International Conference on Learning Representations, ICLR.

[17] Chung S.W., Han S.S., Lee J.W., Oh K.S., Kim N.R., Yoon J.P., Kim J.Y., Moon S.H., Kwon J., Lee H.J. (2018). Automated detection and classification of the proximal humerus fracture by using deep learning algorithm. Acta Orthop..

[18] Kim D.H., MacKinnon T. (2018). Artificial intelligence in fracture detection: Transfer learning from deep convolutional neural networks. Clin. Radiol..

[19] Gan K., Xu D., Lin Y., Shen Y., Zhang T., Hu K., Zhou K., Bi M., Pan L., Wu W. (2019). Artificial intelligence detection of distal radius fractures: A comparison between the convolutional neural network and professional assessments. Acta Orthop..

[20] Kitamura G. (2020). Deep learning evaluation of pelvic radiographs for position, hardware presence, and fracture detection. Eur. J. Radiol..

[21] Cheng C.T., Wang Y., Chen H.W., Hsiao P.M., Yeh C.N., Hsieh C.H., Miao S., Xiao J., Liao C.H., Lu L. (2021). A scalable physician-level deep learning algorithm detects universal trauma on pelvic radiographs. Nat. Commun..

[22] Jones R.M., Sharma A., Hotchkiss R., Sperling J.W., Hamburger J., Ledig C., O’Toole R., Gardner M., Venkatesh S., Roberts M.M. (2020). Assessment of a deep-learning system for fracture detection in musculoskeletal radiographs. Npj Digit. Med..

[23] Buhrmester V., Münch D., Arens M. (2019). Analysis of explainers of black box deep neural networks for computer vision: A survey. arXiv.

[24] Tobler P., Cyriac J., Kovacs B.K., Hofmann V., Sexauer R., Paciolla F., Stieltjes B., Amsler F., Hirschmann A. (2021). AI-based detection and classification of distal radius fractures using low-effort data labeling: Evaluation of applicability and effect of training set size. Eur. Radiol..

[25] Raisuddin A.M., Vaattovaara E., Nevalainen M., Nikki M., Järvenpää E., Makkonen K., Pinola P., Palsio T., Niemensivu A., Tervonen O. (2021). Critical evaluation of deep neural networks for wrist fracture detection. Sci. Rep..

